# Intraosseous vs Intravenous Access for Epinephrine in Pediatric Out-of-Hospital Cardiac Arrest

**DOI:** 10.1001/jamanetworkopen.2025.17291

**Published:** 2025-06-25

**Authors:** Masashi Okubo, Sho Komukai, Junichi Izawa, SunHee Chung, Cameron Dezfulian, Francis X. Guyette, Joshua R. Lupton, Christian Martin-Gill, Sylvia Owusu-Ansah, Sriram Ramgopal, Clifton W. Callaway

**Affiliations:** 1 Department of Emergency Medicine, University of Pittsburgh School of Medicine, Pittsburgh, Pennsylvania; 2 Division of Biomedical Statistics, Department of Integrated Medicine, Osaka University, Graduate School of Medicine, Osaka, Japan; 3 Division of Intensive Care Medicine, Okinawa Prefectural Chubu Hospital, Okinawa, Japan; 4 Department of Preventive Services, Kyoto University Graduate School of Public Health, Kyoto, Japan; 5 Department of Emergency Medicine, Oregon Health and Science University, Portland; 6 Division of Pediatric Critical Care Medicine, Department of Pediatrics, Baylor College of Medicine, Texas Children’s Hospital, Houston; 7 Division of Emergency Medicine, University of Pennsylvania Medical Center Children’s Hospital of Pittsburgh, Pittsburgh; 8 Division of Emergency Medicine, Ann and Robert H. Lurie Children’s Hospital of Chicago, Northwestern University Feinberg School of Medicine, Chicago, Illinois

## Abstract

**Importance:**

While epinephrine is commonly administered in children with out-of-hospital cardiac arrest (OHCA) via an intraosseous (IO) or intravenous (IV) route, the optimal route of epinephrine delivery is unclear.

**Objective:**

To evaluate the association between the route of epinephrine administration (IO or IV) and patient outcomes after pediatric OHCA.

**Design, Setting, and Participants:**

Retrospective cohort study of pediatric patients (aged <18 years) with nontraumatic OHCA treated by emergency medical services who received prehospital epinephrine either via an IO or IV route. Patients were included in the Resuscitation Outcomes Consortium Epidemiologic Registry, a prospective OHCA registry at 10 sites in the US and Canada from April 2011 to June 2015. Data analysis was performed from May 2024 to April 2025.

**Exposure:**

Epinephrine administration route: IO or IV route.

**Main Outcomes and Measures:**

The primary outcome was survival to hospital discharge. The secondary outcome was return of spontaneous circulation (ROSC) before hospital arrival. Propensity scores were calculated and inverse probability of treatment weighting (IPTW) was performed with stabilized weights to control imbalances in measured patient demographics, cardiac arrest characteristics, and bystander and prehospital interventions.

**Results:**

Of 739 eligible patients (median [IQR] age, 1 [0-11] years), 449 (60.8%) were male. Epinephrine was administered via an IO route for 535 (72.4%) and via an IV route for 204 (27.6%) patients. In the IPTW pseudopopulation (740 weighted cases), there was no significant difference in survival to hospital discharge (IO epinephrine: 28 of 528 patients [5.3%] vs IV epinephrine: 12 of 212 patients [5.7%]; risk ratio [RR], 0.92; 95% CI, 0.41-2.07) or prehospital ROSC (IO epinephrine: 76 of 528 patients [14.4%] vs IV epinephrine: 46 of 212 patients [21.7%]; RR, 0.66; 95% CI, 0.42-1.03) between the IO and IV epinephrine groups.

**Conclusions and Relevance:**

In this retrospective cohort study of pediatric patients with OHCA in the US and Canada, the route of epinephrine administration was not associated with survival to hospital discharge or prehospital ROSC. This may support the practice of administering epinephrine via IO or IV route.

## Key Points

**Question:**

Is an intraosseous vs intravenous route for epinephrine administration associated with a difference in survival to hospital discharge among pediatric patients with out-of-hospital cardiac arrest?

**Findings:**

In this cohort study of 739 children with out-of-hospital cardiac arrest using propensity scores and inverse probability of treatment weighting, there was no association between the route of epinephrine administration and survival to hospital discharge.

**Meaning:**

These findings may support a practice of administering epinephrine via either an intraosseous or intravenous route for pediatric patients with out-of-hospital cardiac arrest.

## Teaser

This cohort study evaluates the association between intraosseous vs intravenous epinephrine administration and patient outcomes after pediatric out-of-hospital cardiac arrest (OHCA).

## Introduction

Out-of-hospital cardiac arrest (OHCA) is a substantial public health issue among children, annually affecting 7000 to 23 000 individuals in the US.^1,2^ Only 13.2% of children with OHCA treated by emergency medical services (EMS) survive to hospital discharge, and further efforts are needed to improve their outcomes.^2^

Epinephrine, the cornerstone of cardiac arrest pharmacotherapeutic agents, is commonly used for children with OHCA in prehospital settings, with 65.3% to 74.1% of EMS-treated pediatric OHCAs receiving intraosseous (IO) or intravenous (IV) epinephrine.^3,4^ The 2020 American Heart Association (AHA) Guidelines for Cardiopulmonary Resuscitation and Emergency Cardiovascular Care state that “for pediatric patients in any setting, it is reasonable to administer epinephrine.”^5^ While IO access use has increased and may expedite epinephrine administration due to a higher success rate than IV access,^6,7^ the optimal route of epinephrine administration is unknown.

In 2020, the International Liaison Committee on Resuscitation (ILCOR) published a systematic review and meta-analysis on IO vs IV drug administration for patients of any age during cardiac arrest but identified no pediatric studies.^8^ The pooled estimates of the included adult studies in the meta-analysis favored IV access, with very low certainty of evidence.^8^ In 2024, 3 clinical trials compared an IO-first vs an IV-first strategy for adult OHCAs, and none found a significant difference in survival between the IO- and IV-first strategies.^9,10,11^ However, children were not included in these trials. Therefore, in the pediatric population, further research on the optimal route of epinephrine administration is warranted. Our objective was to evaluate the association between the route of epinephrine administration (ie, IO or IV) and patient outcomes after pediatric OHCA.

## Methods

### Study Design and Setting

We performed a secondary analysis using the Resuscitation Outcomes Consortium (ROC) data. This clinical research network conducted clinical trials in OHCA at 10 regional coordinating sites in the US and Canada.^12,13^ The ROC Epidemiologic Registry-Cardiac Arrest contains information on consecutive patients with nontraumatic OHCA presenting to the study sites from 2011 to 2015. The dataset included patient demographics, cardiac arrest characteristics, bystander and EMS interventions, and patient outcomes. Additional details of the ROC data are provided in the eMethods in Supplement 1. We obtained the publicly available, deidentified ROC dataset from the National Heart, Lung, and Blood Institute Biologic Specimen and Data Repository Information Coordinating Center. The institutional review board at the University of Pittsburgh deemed this study exempt from regulations related to human participant research. We followed the Strengthening the Reporting of Observational Studies in Epidemiology (STROBE) reporting guideline.

### Study Participants

We included pediatric patients (aged <18 years) with EMS-treated nontraumatic OHCA who received epinephrine. EMS-treated OHCA was defined as resuscitation attempts with chest compressions by an EMS clinician and/or shock delivery with an external defibrillator by a bystander or EMS clinician.^13^ To focus on patients for whom IO or IV was the primary route for initial drug delivery, we excluded patients for whom epinephrine was administered via the endotracheal route, via both IO and IV routes, via an unknown route, via an IO route with failed IV access, or via an IV route with failed IO access. We also excluded patients who received vasopressin, had first return of spontaneous circulation (ROSC) before epinephrine administration, or who had missing data about covariates or survival to hospital discharge.

### Exposure and Outcomes

The main exposure was the epinephrine administration route: IO or IV route. The primary outcome was survival to hospital discharge. The secondary outcomes were prehospital ROSC, defined as any ROSC before hospital arrival, and time to prehospital ROSC. For the time to prehospital ROSC, we defined 2 time 0 points: epinephrine administration and advanced life support (ALS) clinician arrival (eMethods in Supplement 1).

### Statistical Analyses

We reported patient demographics, cardiac arrest characteristics, and bystander and EMS interventions, stratified by the route of epinephrine administration with standardized differences. To compare an average treatment effect, we calculated propensity scores (PS) and performed inverse probability of treatment weighting (IPTW) with stabilized weights.^14,15,16,17,18^ PS was generated using logistic regression, with IO epinephrine administration as the dependent variable and age, sex, location of arrest, witness status, initial rhythm, bystander cardiopulmonary resuscitation (CPR), advanced airway placement before epinephrine administration, and interval from dispatch to EMS clinician arrival on scene as covariates. These covariates were chosen based on the known association with the outcomes, biological plausibility, and adequate ascertainment in the dataset.^4,5,19,20^

With the PS, we created a pseudopopulation using IPTW with stabilized weights to control imbalances in the covariates between patients who received IO and IV epinephrine. We assessed the covariate imbalances using a standardized mean difference (SMD) and regarded SMDs less than 0.25 as well-balanced.^21^ Using the pseudopopulation, we fitted generalized linear regression models with Poisson distribution and log link function, which were estimated by quasi-likelihood method, to evaluate the associations of the route of epinephrine administration with survival and ROSC, reporting the risk ratios (RR) with 95% CIs. The variance was evaluated by the sandwich variance estimation.^22^

To compare the difference in time with ROSC, we used Kaplan-Meier estimates to construct cumulative proportion curves of patients pending ROSC over time in the pseudopopulation, grouped by the routes of epinephrine administration. We assessed the between-group difference in time to ROSC using the log-rank test. We fitted Cox regression models with a sandwich variance estimator and reported the hazard ratio (HR) with 95% CI. We conducted 2 time-to-event analyses using 2 time 0 points: epinephrine administration and ALS clinician arrival (eMethods in Supplement 1).

Additionally, we conducted a stratified analysis by age groups (<1 year [infants], 1-9 years [prepuberty children], or ≥10 years [postpuberty adolescents]).^23^ In each age group, we calculated PS and repeated IPTW with stabilized weights.

As a sensitivity analysis, we included those who received epinephrine with either failed IO or IV access and repeated the identical analyses (eMethods in Supplement 1). Because multiple comparisons could potentially cause a type I error, we considered the findings of the stratified and sensitivity analyses as exploratory. All statistical analyses were performed with R software, version 4.1.1 (R Project for Statistical Computing). All tests were 2-tailed and *P* values less than .05 were regarded as significant. Data analysis was performed from May 2024 to April 2025.

## Results

**Figure 1.**
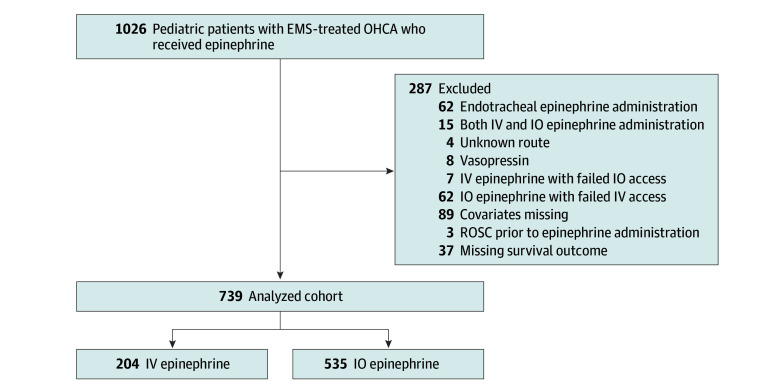
Patient Flow EMS indicates emergency medical services; IO, intraosseous; IV, intravenous; OHCA, out-of-hospital cardiac arrest; ROSC, return of spontaneous circulation.

Seven hundred and thirty-nine patients were eligible for our study (Figure 1). The median (IQR) age was 1 (0-11) year, and 449 patients (60.8%) were male. A total of 535 patients (72.4%) received IO epinephrine, whereas 204 (27.6%) received IV epinephrine. Baseline patient characteristics in the IO and IV epinephrine groups are presented in Table 1. The median (IQR) age was 0 (0-3) years in the IO group and 14 (7-16) years in the IV group. The median (IQR) intervals from ALS clinician arrival to epinephrine administration were 9.0 (6.4-12.3) minutes in the IO group and 8.0 (5.9-11.9) minutes in the IV group, respectively.

**Table 1.** Characteristics of Pediatric Patients With Out-of-Hospital Cardiac Arrest Stratified by the Route of Epinephrine Administration

Characteristic	Patients, No. (%)			Standardized mean difference^a^
	All (N = 739)	Intraosseous epinephrine (n = 535)	Intravenous epinephrine (n = 204)	
Patient demographics				
Age, median (IQR), y	1 (0-11)	0 (0-3)	14 (7-16)	1.590
Sex				
Male	449 (60.8)	314 (58.7)	135 (66.2)	0.155
Female	290 (39.2)	221 (41.3)	69 (33.8)	
Arrest characteristics				
Location				
Private location	663 (89.7)	504 (94.2)	159 (77.9)	0.483
Public location	76 (10.3)	31 (5.8)	45 (22.1)	
Witness status				
Bystander witnessed	149 (20.2)	99 (18.5)	50 (24.5)	0.174
EMS witnessed	28 (3.8)	18 (3.4)	10 (4.9)	
Unwitnessed	562 (76.0)	418 (78.1)	144 (70.6)	
Initial rhythm				
Shockable	42 (5.7)	19 (3.6)	23 (11.3)	0.298
Nonshockable	697 (94.3)	516 (96.4)	181 (88.7)	
Bystander intervention				
Bystander CPR				
Presence	347 (47.0)	252 (47.1)	95 (46.6)	0.011
Absence	392 (53.0)	283 (52.9)	109 (53.4)	
EMS intervention				
Advanced airway management before epinephrine administration				
Presence	132 (17.9)	99 (18.5)	33 (16.2)	0.528
Absence	607 (82.1)	436 (81.5)	171 (83.8)	
Interval from dispatch to EMS clinician arrival, median (IQR), min	5.1 (3.9-6.5)	5.0 (3.9-6.4)	5.3 (4.0-6.9)	0.177
Interval from dispatch to epinephrine, median (IQR), min	16.3 (13.0-20.2)	16.2 (13.0-19.9	16.6 (13.0-21.0)	0.211
Interval from ALS clinician arrival to epinephrine, median (IQR), min	8.8 (6.2-12.1)	9.0 (6.4-12.3)	8.0 (5.9-11.9)	0.037

Abbreviations: ALS, advanced life support; CPR, cardiopulmonary resuscitation; EMS, emergency medical services.

^a^ Standardized mean difference between intraosseous and intravenous epinephrine groups.

Using IPTW with stabilized weights, we created a pseudopopulation including 528 cases in the IO epinephrine group and 212 cases in the IV epinephrine group (Table 2). We successfully balanced all covariates between the IO and IV groups, with all SMDs less than 0.25. We present the distribution of PS (eFigure 1 in Supplement 1), the association between PS and weighting (eFigure 2 in Supplement 1), and the association between patient age and weighting (eFigure 3 in Supplement 1). The median (IQR) interval from ALS clinician arrival to epinephrine administration was 8.4 (6.1-12.0) minutes in the IO and 9.4 (6.0-12.9) minutes in the IV groups, respectively.

**Table 2.** Characteristics of Pediatric Patients With Out-of-Hospital Cardiac Arrest After Inverse Probability of Treatment Weighting

Characteristic	Patients, %		Standardized mean difference
	Intraosseous epinephrine (n = 528)	Intravenous epinephrine (n = 212)	
Patient demographics			
Age, median (IQR), y	1 (0-10)	1 (0-10)	0.009
Sex			
Male	60.1	58.4	0.035
Female	39.9	41.6	
Arrest characteristics			
Location			
Private location	92.0	91.7	0.013
Public location	8.0	8.3	
Witness status			
Bystander witnessed	19.3	20.2	0.06
EMS witnessed	3.4	2.4	
Unwitnessed	77.3	77.3	
Initial rhythm			
Shockable	4.0	4.1	0.004
Nonshockable	96.0	95.9	
Bystander intervention			
Bystander CPR			0.148
Presence	45.3	38.0	
Absence	54.7	62.0	
EMS intervention			
Advanced airway management before epinephrine administration			
Presence	17.5	20.8	0.084
Absence	82.5	79.2	
Interval from dispatch to EMS clinician arrival, median (IQR), minutes	5.0 (3.9-6.6)	5.2 (3.9-6.3)	0.020
Interval from dispatch to epinephrine, median (IQR), min^a^	16.1 (12.8-19.8)	17.6 (13.0-21.5)	0.189
Interval from ALS clinician arrival to epinephrine, median (IQR), min^a^	8.4 (6.1-12.0)	9.4 (6.0-12.9)	0.149

Abbreviations: ALS, advanced life support; CPR, cardiopulmonary resuscitation; EMS, emergency medical services.

^a^ Not included in the propensity score model as a covariate.

In the weighted analysis, the likelihood of survived to hospital discharge did not differ between patients who received IO and an IV epinephrine (IO epinephrine: 28 of 528 patients [5.3%] vs IV epinephrine: 12 of 212 patients [5.7%]; RR, 0.92; 95% CI, 0.41-2.07) (Table 3). The likelihood of prehospital ROSC also did not differ between the IO and IV epinephrine groups (IO epinephrine: 76 of 528 patients [14.4%] vs IV epinephrine: 46 of 212 patients [21.7%]; RR, 0.66; 95% CI, 0.42-1.03).

**Table 3.** Outcomes in Inverse Probability of Treatment Weights Analyses

Outcome	Patients, No./total No. (%)		Risk ratio (95% CI)
	Intraosseous epinephrine	Intravenous epinephrine	
All patients			
Survival to hospital discharge	28/528 (5.3)	12/212 (5.7)	0.92 (0.41-2.07)
Prehospital ROSC	76/528 (14.4)	46/212 (21.7)	0.66 (0.42-1.03)
Stratified analyses			
Age <1 y			
Survival to hospital discharge	4/271 (1.5)	0/21	NA^a^
Prehospital ROSC	17/271 (6.3)	1/21 (4.7)	1.10 (0.16-7.80)
Age 1-9 y			
Survival to hospital discharge	12/196 (6.1)	2/43 (4.7)	1.28 (0.21-7.68)
Prehospital ROSC	23/196 (11.7)	12/43 (27.9)	0.40 (0.18-0.88)
Age ≥10 y			
Survival to hospital discharge	7/64 (10.9)	21/143 (14.7)	0.72 (0.30-1.72)
Prehospital ROSC	19/64 (29.7)	59/143 (41.3)	0.73 (0.45-1.18)

Abbreviations: NA, not applicable; ROSC, return of spontaneous circulation.

^a^ The model did not converge.

**Figure 2.**
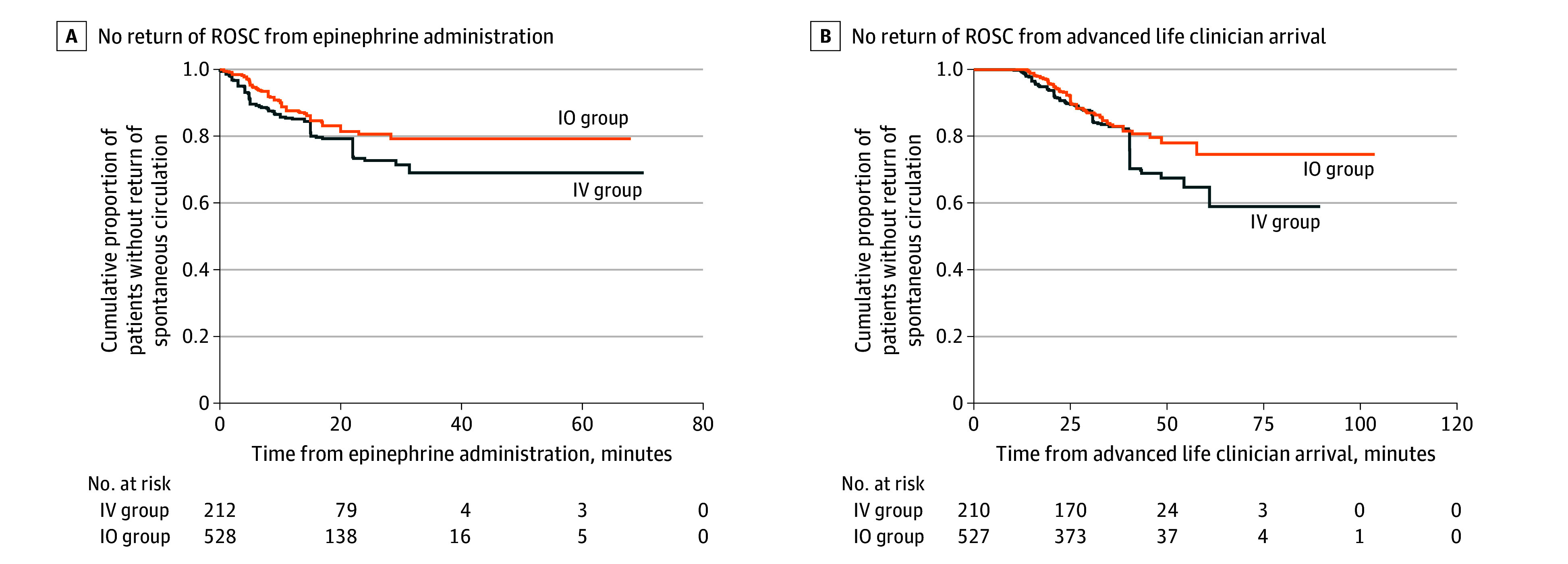
Cumulative Proportion of Patients Without Return of Spontaneous Circulation (ROSC) Over Time From Epinephrine Administration and Advanced Life Clinician Arrival in the Weighted Population, Stratified by the Route of Epinephrine Administration A, Log-rank *P* = .29. The hazard ratio for the IO group was 0.71 (95% CI, 0.43-1.20). B, Log-rank *P* = .41. The hazard ratio for the IO group was 0.77 (95% CI, 0.47-1.28). IO indicates intraosseous; IV, intravenous.

We did not detect a difference in time to ROSC after epinephrine administration by the vascular accesses using Kaplan-Meier estimates (log-rank *P* = .29) or HR (0.71; 95% CI, 0.43-1.20). Using ALS clinician arrival as the time 0, the results were similar (Figure 2B).

In the stratified analyses, the age groups of less than 1 year (infants), 1 to 9 years (prepuberty children), and 10 or more years (postpuberty adolescents) in the original subcohorts included 293 (IO group, 271 patients; IV group, 22 patients), 238 (IO group, 197 patients; IV group, 41 patients), and 208 (IO group, 67 patients; IV group, 141 patients) patients, respectively. The median (IQR) intervals from ALS clinician arrival to epinephrine administration in the original subcohorts were 9.9 (7.0-13.0) minutes in the IO group and 9.2 (6.3-11.9) minutes in the IV group in infants; 8.1 (6.4-11.4) minutes in the IO group and 9.4 (7.0-12.3) minutes in the IV group in prepuberty children; and 7.1 (5.2-11.8) minutes in the IO group and 7.6 (5.4-11.7) minutes in the IV group in postpuberty adolescents, respectively. In the IPTW subcohorts, all covariates were well-balanced between the IO and IV epinephrine groups, with all SMDs in the weighted cohort less than 0.25, except bystander CPR in prepuberty children (eTables 1-3 in Supplement 1). In infants, the model for survival to hospital discharge did not converge since the outcome event was 0 in the IV epinephrine group (Table 3). The survival did not differ between the IO and IV epinephrine groups in prepuberty children (RR, 1.28; 95% CI, 0.21-7.68) or postpuberty adolescents (RR, 0.72; 95% CI, 0.30-1.72). The prehospital ROSC did not differ between the IO and IV epinephrine groups in infants (RR, 1.10; 95% CI, 0.16-7.80) or postpuberty adolescents (RR, 0.73; 95% CI, 0.45-1.18). In contrast, in prepuberty children, the likelihood of prehospital ROSC was lower in the IO group (RR, 0.40; 95% CI, 0.18-0.88). Time to ROSC from epinephrine administration or ALS clinician arrival did not differ between the IO and IV epinephrine groups across all age groups (eFigures 4-6 in Supplement 1), except that the cumulative proportion of patients without ROSC after epinephrine administration was higher in the IO epinephrine group among prepuberty children (eFigure 5 in Supplement 1).

In the sensitivity analysis, which included those who received epinephrine with either failed IO or IV access, similar findings were observed as in the primary analysis except that the proportion of patients who had prehospital ROSC was lower in the IO epinephrine group (RR, 0.65; 95% CI, 0.42-0.99) (eTable 4 and eFigure 7 in Supplement 1).

## Discussion

In our analysis of a large North American OHCA dataset using IPTW, we observed that epinephrine administration via an IO route was not associated with survival to hospital discharge, prehospital ROSC, or time to ROSC, compared with epinephrine administration via an IV route. The findings were similar in the sensitivity analysis. We also found that the majority of patients (72.4%) received IO epinephrine, and the route of epinephrine administration was correlated with patient age; that is, younger patients tended to receive IO epinephrine, which might reflect EMS clinicians’ preference and/or an expected attainability of IO access.

As the 2020 ILCOR systematic review and meta-analyses on IO vs IV drug administration during cardiac arrest identified no pediatric studies,^8^ prior knowledge about the optimal route of epinephrine administration is markedly limited for children. The meta-analyses of adult studies showed that IO access was associated with the decreased likelihood of survival to hospital discharge (odds ratio [OR], 0.71; 95% CI, 0.63-0.79) with very low certainty of evidence due to confounding by indication and resuscitation time bias.^8,24^ A retrospective analysis of the French national OHCA registry of 603 prepubescent patients (male aged <12 years and female aged <10 years) in 2021 demonstrated that epinephrine administration via an IO route was not associated with ROSC (OR, 1.00; 95% CI, 0.52-1.93) nor survival to hospital discharge (OR, 1.00; 95% CI, 0.20-5.08), compared with an IV route using PS matching.^25^ The PS-matched cohort included 202 patients, which limited the sample size and resulted in broad 95% CIs.

Another observational study of the ROC in 2021 reported that, among 761 pediatric patients (aged <18 years) with OHCA, IO access was associated with a decreased likelihood of survival to hospital discharge (OR, 0.46; 95% CI, 0.21-0.98), compared with an IV access using a logistic regression model.^7^ It is essential to note the differences between the prior study and our analysis. First, the prior study assessed the association of vascular access (ie, IO or IV) with survival, not the association of the route of epinephrine administration (IO or IV) with survival. In the prior study, 9.0% in IO and 23.1% in IV groups did not receive epinephrine, whereas all patients in our analysis received IO or IV epinephrine.^7^ Second, the prior study included those who had IO access with failed IV access and those who had IV access with failed IO access, whereas in our primary analysis, we excluded those who received epinephrine via an alternative route to evaluate the initially attempted route of epinephrine administration. Third, the difference in the statistical approaches resulted in different estimands. The logistic regression model in the prior study provided the estimated conditional effect of IO access within the covariates’ strata, whereas the IPTW in ours provided the estimated marginal effect (ie, average treatment effect) in the entire study population.^16,26,27^

In 2024, 3 prehospital clinical trials comparing an IO-first vs an IV-first strategy for adult patients with OHCA were published, and all reported that there was no significant difference in survival between the IO-first and the IV-first strategies.^9,10,11^ A cluster randomized clinical trial in Taiwan, the Intraosseous vs Intravenous Vascular Access in Upper Extremity Among Adults With Out-of-Hospital Cardiac Arrest trial, including 1732 adult patients with OHCA, found no differences in survival to hospital discharge (OR, 1.04; 95% CI, 0.76-1.42), favorable functional status at hospital discharge (OR, 1.17; 95% CI, 0.82-1.66), or prehospital ROSC (OR, 0.92; 95% CI, 0.75-1.13) between IO and IV groups.^10^ A multicenter randomized trial in the United Kingdom (UK), the Prehospital Randomised Trial of Medication Route in Out-of-Hospital Cardiac Arrest (PARAMEDIC 3), included 3040 adults in the IO-first and 3042 in the IV-first groups and found no significant difference in survival at 30 days (OR, 0.94; 95% CI, 0.68-1.32) and favorable functional status at hospital discharge (OR, 0.91; 95% CI, 0.57-1.47), while the odds of ROSC at any time was lower in the IO-first group (OR, 0.86; 95% CI, 0.76-0.97).^9^ Another trial comparing the effects of initial attempts at IO vs IV vascular access among 1479 adult patients in Denmark, the Intravenous vs Intraosseous Vascular Access during Out-of-Hospital Cardiac arrest (IVIO) trial, found no differences in survival at 30 days (risk ratio [RR], 1.16; 95% CI, 0.87-1.56), favorable functional status at hospital discharge (RR, 1.16; 95% CI, 0.83-1.62), or sustained ROSC (RR, 1.06; 95% CI, 0.90-1.24).^11^ These trials’ results suggest that vascular access may not affect survival or functional status among adult patients with OHCA.

As a clinical implication, our results may support the current AHA Resuscitation Guidelines’ recommendation of either IO or IV epinephrine administration for pediatric patients with cardiac arrest.^5^ Our results also bring several research implications. First, our neutral results might generate a clinical equipoise that justifies a future clinical trial to compare IO epinephrine vs IV epinephrine for pediatric OHCA. Given the differences in the causes and outcomes of OHCA between adults and children, the recent adult trials’ results may not be applicable to children, and a dedicated pediatric trial would identify an optimal drug administration strategy for children.

Although a subanalysis of the IVIO trial in Denmark also compared tibial IO with humeral IO epinephrine administration strategy and found no differences in sustained ROSC or 30-day survival,^11^ the subanalysis might have been underpowered. The optimal dose and interval of epinephrine administration by the administration routes and sites for pediatric OHCA are not fully investigated and further work is needed.

There are several potential reasons why we did not observe a difference in the association of the routes of epinephrine administration with patient outcomes. First, the sample size in our analysis was limited. The PARAMEDIC 3 in the UK planned to include 15 000 participants (7500 in the IO-first strategy and 7500 in the IV-first strategy) to detect a 1% difference in 30-day survival (3.2% vs 4.2%) with a 2-sided significance level of 5% and power of 90%.^28^ Given the difference in the planned sample sizes of PARAMEDIC 3 and our study, it is likely that our analysis was underpowered.

Second, a prior observational study showed that epinephrine administration via an IO route for OHCA was associated with a shorter time from EMS arrival to the epinephrine administration (5.0; 95% CI, 4.7-5.4 minutes), compared with an IV route (8.8; 95% CI, 6.6-10.9 minutes).^29^ On the other hand, in our study, the median intervals from ALS clinician arrival to epinephrine administration were not different between the IO and IV groups in both original (9.0 vs 8.0 minutes) and IPTW (8.4 vs 9.4 minutes) cohorts, with SMDs less than 0.25. It is possible that in the IO group in our study, other resuscitative interventions were prioritized over the IO access, which could have led to the delayed IO access and neutral results .

### Limitations

This study has limitations. First, the decision of routes for epinephrine administration was made by EMS clinicians, not at random. This could have led to confounding by indication.^30^ In addition, although we adjusted for measured confounding factors, there may be residual confounding factors such as chest compression metrics and patients’ body mass index and underlying comorbidities, which were not available in the dataset. Second, the anatomic location of IO access was unavailable, and we could not evaluate IO epinephrine administration by the location of IO sites. We treated epinephrine administration via an IO route as an average effect of epinephrine administration via any IO sites. Third, the choice of routes for epinephrine administration may have been associated with EMS systems. Since, in the dataset, information about EMS systems was not available, we were unable to account for clustering of patients within EMS systems. Finally, the results may not be applicable to other EMS systems since selected EMS systems were included in ROC based on adherence to performance metrics, ability to conduct trials, and interest in participating in research.

## Conclusions

In this observational study of pediatric OHCA in North America, epinephrine administration via an IO route was not associated with survival to hospital discharge, prehospital ROSC, or time to prehospital ROSC among overall pediatric patients compared with epinephrine administration via an IV route. This may support the practice of administering epinephrine via either IO or IV route.

## Notes

Supplement 1.
eMethods.
**eTable 1. **Characteristics of pediatric patients who were younger than 1 year with out-of-hospital cardiac arrest after inverse probability of treatment weighting **eTable 2.** Characteristics of pediatric patients who were older than or equal to 1 year and younger than or equal to 9 years with out-of-hospital cardiac arrest after inverse probability of treatment weighting **eTable 3. **Characteristics of pediatric patients who were older than or equal to 10 years with out-of-hospital cardiac arrest after inverse probability of treatment weighting **eTable 4. **Outcomes in inverse probability of treatment weights analyses including those who received epinephrine with either failed IO or IV access (sensitivity analysis) **eFigure 1. **Distribution of propensity score, stratified by intraosseous (IO) and intravenous (IV) epinephrine administration **eFigure 2. **Association between propensity score and weighting **eFigure 3.** Association between patient age and weighting **eFigure 4. **Cumulative proportion of patients without return of spontaneous circulation over time from epinephrine administration (A) and advanced life clinician arrival (B) in the weighted population, among who were younger than 1 year, stratified by the route of epinephrine administration **eFigure 5. **Cumulative proportion of patients without return of spontaneous circulation over time from epinephrine administration (A) and advanced life clinician arrival (B) in the weighted population, among who were older than or equal to 1 year and younger than or equal to 9 years, stratified by the route of epinephrine administration **eFigure 6.** Cumulative proportion of patients without return of spontaneous circulation over time from epinephrine administration (A) and advanced life clinician arrival (B) in the weighted population, among who were older than or equal to 10 years, stratified by the route of epinephrine administration **eFigure 7.** Cumulative proportion of patients pending return of spontaneous circulation over time in the weighted population including those who received epinephrine with either failed IO or IV access, stratified by the route of epinephrine administration (sensitivity analysis)

Supplement 2.
Data Sharing Statement
