# Supplementary material for: Intraosseous vs Intravenous Access for Epinephrine in Pediatric Out-of-Hospital Cardiac Arrest
Source: JAMA Netw Open. 2025 Jun 25;8(6):e2517291. doi: 10.1001/jamanetworkopen.2025.17291 (PMC12199053; doi:10.1001/jamanetworkopen.2025.17291)
Supplement: Supplement 1. — eMethods. eTable 1. Characteristics of pediatric patients who were younger than 1 year with out-of-hospital cardiac arrest after inverse probability of treatment weighting eTable 2. Characteristics of pediatric patients who were older than or equal to 1 year and younger than or equal to 9 years with out-of-hospital cardiac arrest after inverse probability of treatment weighting eTable 3. Characteristics of pediatric patients who were older than or equal to 10 years with out-of-hospital cardiac arrest after inverse probability of treatment weighting eTable 4. Outcomes in inverse probability of treatment weights analyses including those who received epinephrine with either failed IO or IV access (sensitivity analysis) eFigure 1. Distribution of propensity score, stratified by intraosseous (IO) and intravenous (IV) epinephrine administration eFigure 2. Association between propensity score and weighting eFigure 3. Association between patient age and weighting eFigure 4. Cumulative proportion of patients without return of spontaneous circulation over time from epinephrine administration (A) and advanced life clinician arrival (B) in the weighted population, among who were younger than 1 year, stratified by the route of epinephrine administration eFigure 5. Cumulative proportion of patients without return of spontaneous circulation over time from epinephrine administration (A) and advanced life clinician arrival (B) in the weighted population, among who were older than or equal to 1 year and younger than or equal to 9 years, stratified by the route of epinephrine administration eFigure 6. Cumulative proportion of patients without return of spontaneous circulation over time from epinephrine administration (A) and advanced life clinician arrival (B) in the weighted population, among who were older than or equal to 10 years, stratified by the route of epinephrine administration eFigure 7. Cumulative proportion of patients pending return of spontaneous circulation over time i [file jamanetwopen-e2517291-s001.pdf]

Supplemental Online Content

Okubo M, Komukai S, Izawa J, et al. Intraosseous vs intravenous access for epinephrine in pediatric out-of-hospital cardiac arrest. *JAMA Netw Open*. 2025;8(6):e2517291. doi:10.1001/jamanetworkopen.2025.17291

eMethods.
eTable 1. Characteristics of pediatric patients who were younger than 1 year with out-of-hospital cardiac arrest after inverse probability of treatment weighting
eTable 2. Characteristics of pediatric patients who were older than or equal to 1 year and younger than or equal to 9 years with out-of-hospital cardiac arrest after inverse probability of treatment weighting
eTable 3. Characteristics of pediatric patients who were older than or equal to 10 years with out-of-hospital cardiac arrest after inverse probability of treatment weighting
eTable 4. Outcomes in inverse probability of treatment weights analyses including those who received epinephrine with either failed IO or IV access (sensitivity analysis)
eFigure 1. Distribution of propensity score, stratified by intraosseous (IO) and intravenous (IV) epinephrine administration
eFigure 2. Association between propensity score and weighting
eFigure 3. Association between patient age and weighting
eFigure 4. Cumulative proportion of patients without return of spontaneous circulation over time from epinephrine administration (A) and advanced life clinician arrival (B) in the weighted population, among who were younger than 1 year, stratified by the route of epinephrine administration
eFigure 5. Cumulative proportion of patients without return of spontaneous circulation over time from epinephrine administration (A) and advanced life clinician arrival (B) in the weighted population, among who were older than or equal to 1 year and younger than or equal to 9 years, stratified by the route of epinephrine administration
eFigure 6. Cumulative proportion of patients without return of spontaneous circulation over time from epinephrine administration (A) and advanced life clinician arrival (B) in the weighted population, among who were older than or equal to 10 years, stratified by the route of epinephrine administration
eFigure 7. Cumulative proportion of patients pending return of spontaneous circulation over time in the weighted population including those who received epinephrine with either failed IO or IV access, stratified by the route of epinephrine administration (sensitivity analysis)

This supplemental material has been provided by the authors to give readers additional information about their work.

© 2025 Okubo M et al. *JAMA Network Open*.

eMethods

Resuscitation Outcomes Consortium (ROC) Epidemiologic Registry (Epistry)-Cardiac Arrest

A case was considered an out-of-hospital cardiac arrest (OHCA), and thus eligible for the registry, if: (1) emergency medical services (EMS) performed chest compressions; (2) if a shock was delivered by an external defibrillator (by layperson or EMS personnel); or, (3) if the patient was identified as pulseless and no treatments were attempted by EMS personnel (cases in this last group were classified as “EMS-untreated”).[1] The ROC was a clinical research network that examined the treatment and subsequent outcomes of patients with OHCA across 10 regional coordinating sites in the United States and Canada.[1,2] The ROC Epistry collected data from April 2011 to June 2015. To ensure the data integrity, the ROC instituted several quality assurance protocols including periodic training sessions for research teams in data collection and variable definitions, reviews of randomly selected records at each site by the data coordinating center, and logic checks of data element range and consistency in both the online data entry forms and the batch upload process.[1] Moreover, the data coordinating center undertook annual site visits to further review a portion of entered records, data capture process, and site-specific mechanisms for quality assurance.[1] The ROC used several quality assurance plans including periodic training of research teams in data collection and variable definitions, review of randomly selected records at each site by the data coordinating center, and logic checks of data element range and consistency in both the web-based data entry forms and the batch upload process.[1] Additionally, the data coordinating center conducted annual site visits to further review a portion of entered records, data capture process, and site-specific mechanisms for quality assurance.[1]

Outcome: Time to return of spontaneous circulation (ROSC)

© 2025 Okubo M et al. *JAMA Network Open*.

Using a target trial emulation framework,[3] the time zero for the time to ROSC should ideally be the point when advanced life support (ALS) clinicians attempted either vascular access (IO or IV), however, such a time variable was not available. We, therefore, used the time points of epinephrine administration and ALS clinician arrival as the surrogate of the time when ALS clinicians attempted either vascular access.

Sensitivity Analysis

Primary analysis excluded those who received epinephrine via an IO route with failed IV access or via an IV route with failed IO access. Since the ROC dataset did not have information on the timing of the failed intraosseous (IO) or intravenous (IV) access and we were unable to determine whether the failed vascular accesses occurred before or after a successful alternative vascular access, we might have excluded not only those who received epinephrine via a rescue route after a failed IO or IV access but also those who received IO or IV epinephrine and subsequently had a failed alternative vascular access after epinephrine administration. For example, for a patient who received IO epinephrine with a failed IV access, we were unable to differentiate whether the patient received IO epinephrine, achieved return of spontaneous circulation (ROSC), and had a failed IV access after ROSC or the patient had a failed IV access, received IO epinephrine via the rescue route (IO), and achieved ROSC. We, therefore, conducted a sensitivity analysis, including those who received epinephrine with either failed IO or IV access.

1. Morrison LJ, Nichol G, Rea TD, et al. Rationale, development and implementation of the Resuscitation Outcomes Consortium Epistry-Cardiac Arrest. *Resuscitation.* 2008;78(2):161-169.
2. Davis DP, Garberson LA, Andrusiek DL, et al. A descriptive analysis of Emergency Medical Service Systems participating in the Resuscitation Outcomes Consortium (ROC) network. *Prehosp Emerg Care.* 2007;11(4):369-382.
3. Hernán MA, Wang W, Leaf DE. Target Trial Emulation: A Framework for Causal Inference From Observational Data. *Jama.* 2022;328(24):2446-2447.

© 2025 Okubo M et al. *JAMA Network Open*.

eTable 1. Characteristics of pediatric patients who were younger than 1 year with out-of-hospital cardiac arrest after inverse probability of treatment weighting

	Intraosseous epinephrine (n=271)	Intravenous epinephrine (n=21)	Standardized mean difference
**Patient demographics**			
Age, median (IQR), years	0 (0-0)	0 (0-0)	<0.001
Sex, %			0.097
Male	60.1	64.8	
Female	39.9	35.2	
**Arrest characteristics**			
Location, %			0.058
Private location	95.9	97.0	
Public location	4.1	3.0	
Witness status, %			0.170
Bystander witnessed	13.6	12.7	
EMS witnessed	1.4	0.0	
Unwitnessed	85.0	87.3	
Initial rhythm, %			0.186
Shockable	1.7	0.0	
Nonshockable	98.3	100.0	
**Bystander intervention**			
Bystander CPR, %			0.034
Presence	49.5	51.2	
Absence	50.5	48.8	
**EMS intervention**			
Advanced airway management before epinephrine administration, %			0.057
Presence	15.0	13.0	

© 2025 Okubo M et al. *JAMA Network Open*.

Absence	85.0	87.0	
Interval from dispatch to EMS clinician arrival, median (IQR), minutes	4.9 (3.8-6.3)	5.3 (3.8-6.0)	0.003
Interval from dispatch to epinephrine, median (IQR), minutes[a]	16.3 (13.4-20.0)	17.5 (11.5-20.6)	0.001
Interval from ALS clinician arrival to epinephrine, median (IQR), minutes[a]	9.9 (7.0-12.9)	8.6 (6.0-13.9)	0.162

[a] Not included in the propensity score model as a covariate.

Abbreviations: ALS, advanced life support; CPR, cardiopulmonary resuscitation; EMS, emergency medical services; IQR, interquartile range

© 2025 Okubo M et al. *JAMA Network Open*.

eTable 2. Characteristics of pediatric patients who were older than or equal to 1 year and younger than or equal to 9 years with out-of-hospital cardiac arrest after inverse probability of treatment weighting

	Intraosseous epinephrine (n=196)	Intravenous epinephrine (n=43)	Standardized mean difference
**Patient demographics**			
Age, median (IQR), years	2 (1-5)	2 (1-4)	0.091
Sex, %			0.136
Male	59.3	52.6	
Female	40.7	47.4	
**Arrest characteristics**			
Location, %			0.081
Private location	91.7	93.8	
Public location	8.3	6.2	
Witness status, %			0.190
Bystander witnessed	23.5	31.5	
EMS witnessed	4.7	3.0	
Unwitnessed	71.9	65.5	
Initial rhythm, %			0.120
Shockable	4.2	2.1	
Nonshockable	95.8	97.9	
**Bystander intervention**			
Bystander CPR, %			0.284
Presence	42.5	29.0	
Absence	57.5	71.0	
**EMS intervention**			
Advanced airway management before epinephrine administration, %			0.142
Presence	19.8	25.8	

© 2025 Okubo M et al. *JAMA Network Open*.

Absence	80.2	74.2	
Interval from dispatch to EMS clinician arrival, median (IQR), minutes	5.0 (3.9-6.4)	4.8 (3.9-6.5)	0.009
Interval from dispatch to epinephrine, median (IQR), minutes[a]	15.5 (12.3-19.4)	19.1 (14.9-22.3)	0.418
Interval from ALS clinician arrival to epinephrine, median (IQR), minutes[a]	8.0 (6.4-11.4)	11.1 (7.2-15.0)	0.462

[a] Not included in the propensity score model as a covariate.

Abbreviations: ALS, advanced life support; CPR, cardiopulmonary resuscitation; EMS, emergency medical services; IQR, interquartile range

© 2025 Okubo M et al. *JAMA Network Open*.

e3. Characteristics of pediatric patients who were older than or equal to 10 years with out-of-hospital cardiac arrest after inverse probability of treatment weighting

	Intraosseous epinephrine (n=64)	Intravenous epinephrine (n=143)	Standardized mean difference
**Patient demographics**			
Age, median (IQR), years	14 (12-16)	15 (12-16)	0.023
Sex, %			0.011
Male	61.4	61.9	
Female	38.6	38.1	
**Arrest characteristics**			
Location, %			0.158
Private location	85.9	79.9	
Public location	14.1	20.1	
Witness status, %			0.052
Bystander witnessed	23.3	25.2	
EMS witnessed	5.3	5.9	
Unwitnessed	71.3	69.0	
Initial rhythm, %			0.183
Shockable	7.1	12.5	
Nonshockable	92.9	87.5	
**Bystander intervention**			
Bystander CPR, %			0.032
Presence	44.4	46.0	
Absence	55.6	54.0	
**EMS intervention**			
Advanced airway management before epinephrine administration, %			0.067
Presence	18.4	21.0	

© 2025 Okubo M et al. *JAMA Network Open*.

Absence	81.6	79.0	
Interval from dispatch to EMS clinician arrival, median (IQR), minutes	6.0 (4.4-7.1)	5.7 (4.2-7.2)	0.085
Interval from dispatch to epinephrine, median (IQR), minutes[a]	16.2 (12.7-19.1)	16.7 (12.9-21.1)	0.265
Interval from ALS clinician arrival to epinephrine, median (IQR), minutes[a]	7.1 (5.0-10.5)	7.6 (5.3-11.8)	0.271

[a] Not included in the propensity score model as a covariate.

Abbreviations: ALS, advanced life support; CPR, cardiopulmonary resuscitation; EMS, emergency medical services; IQR, interquartile range

© 2025 Okubo M et al. *JAMA Network Open*.

eTable 4. Outcomes in inverse probability of treatment weights analyses, including those who received epinephrine with either failed intraosseous or intravascular access (Sensitivity analysis)

	Intraosseous epinephrine	Intravenous epinephrine	Risk ratio (95% confidence interval)
All patients, n (%)			
Survival to hospital discharge	32/561 (5.7)	12/215 (5.6)	0.98 (0.46-2.10)
Prehospital ROSC	79/561 (14.1)	46/215 (21.4)	0.65 (0.42-0.9992)

Abbreviations: ROSC, return of spontaneous circulation

© 2025 Okubo M et al. *JAMA Network Open*.

eFigure 1. Distribution of propensity score, stratified by intraosseous (IO) and intravenous (IV) epinephrine administration.

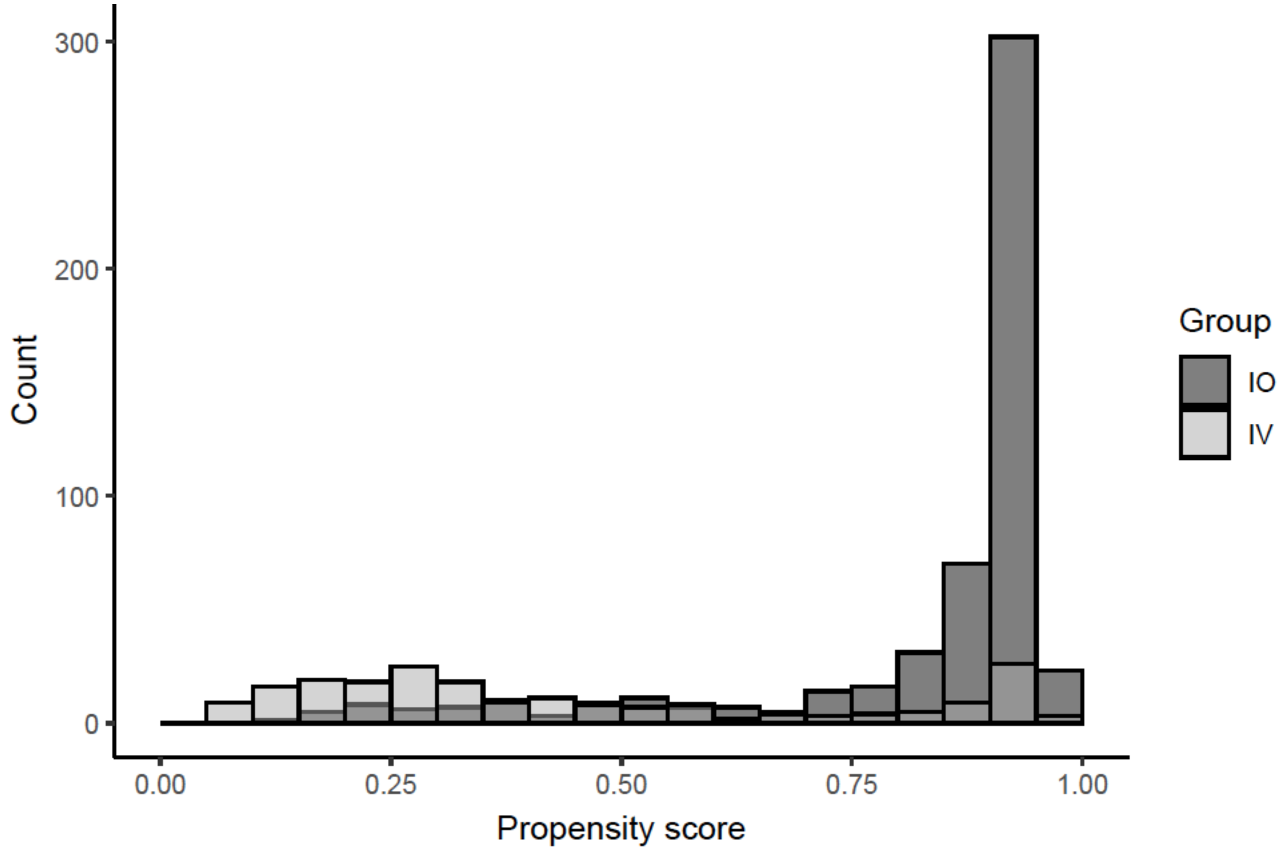

© 2025 Okubo M et al. *JAMA Network Open*.

eFigure 2. Association between propensity score and weighting.
Each dot represents each patient.

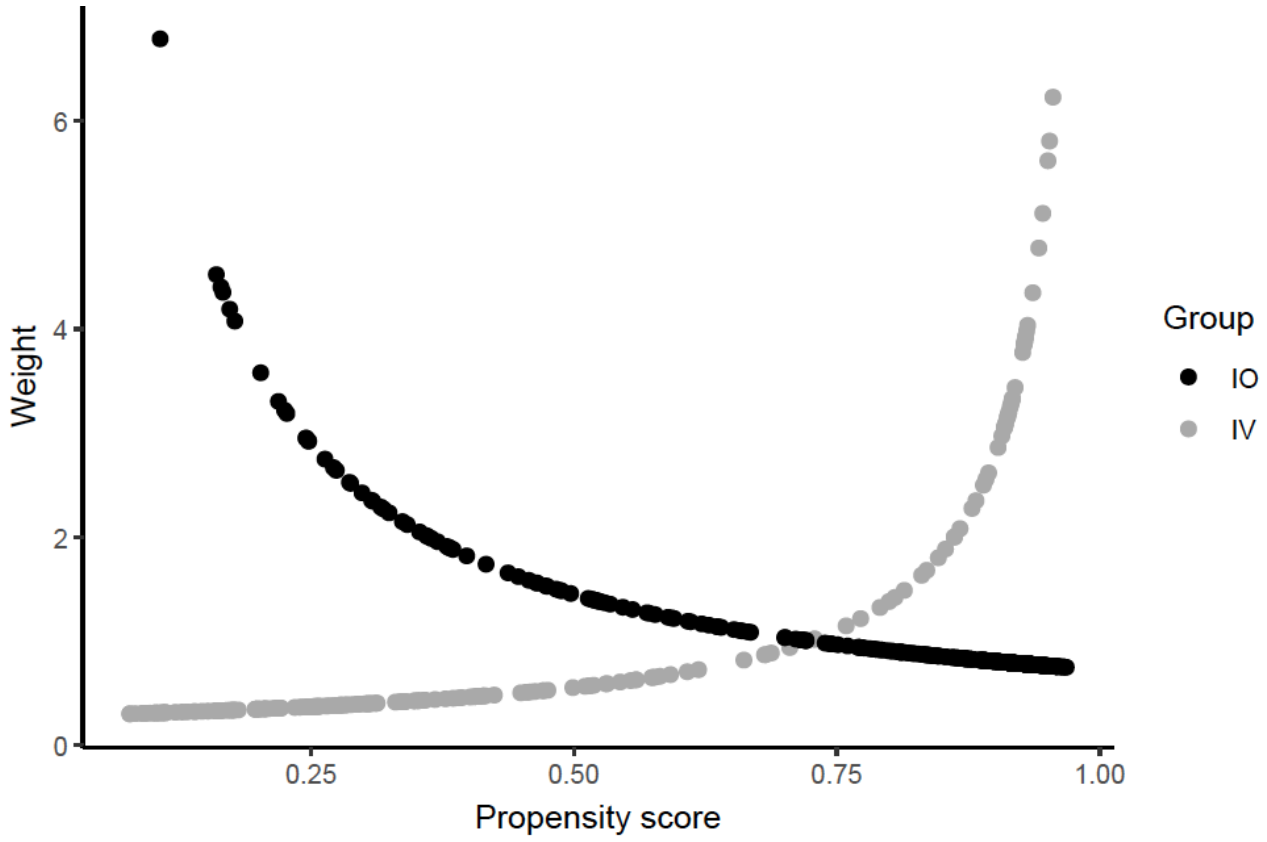

© 2025 Okubo M et al. *JAMA Network Open*.

eFigure 3. Association between patient age and weighting
Each dot represents each patient.

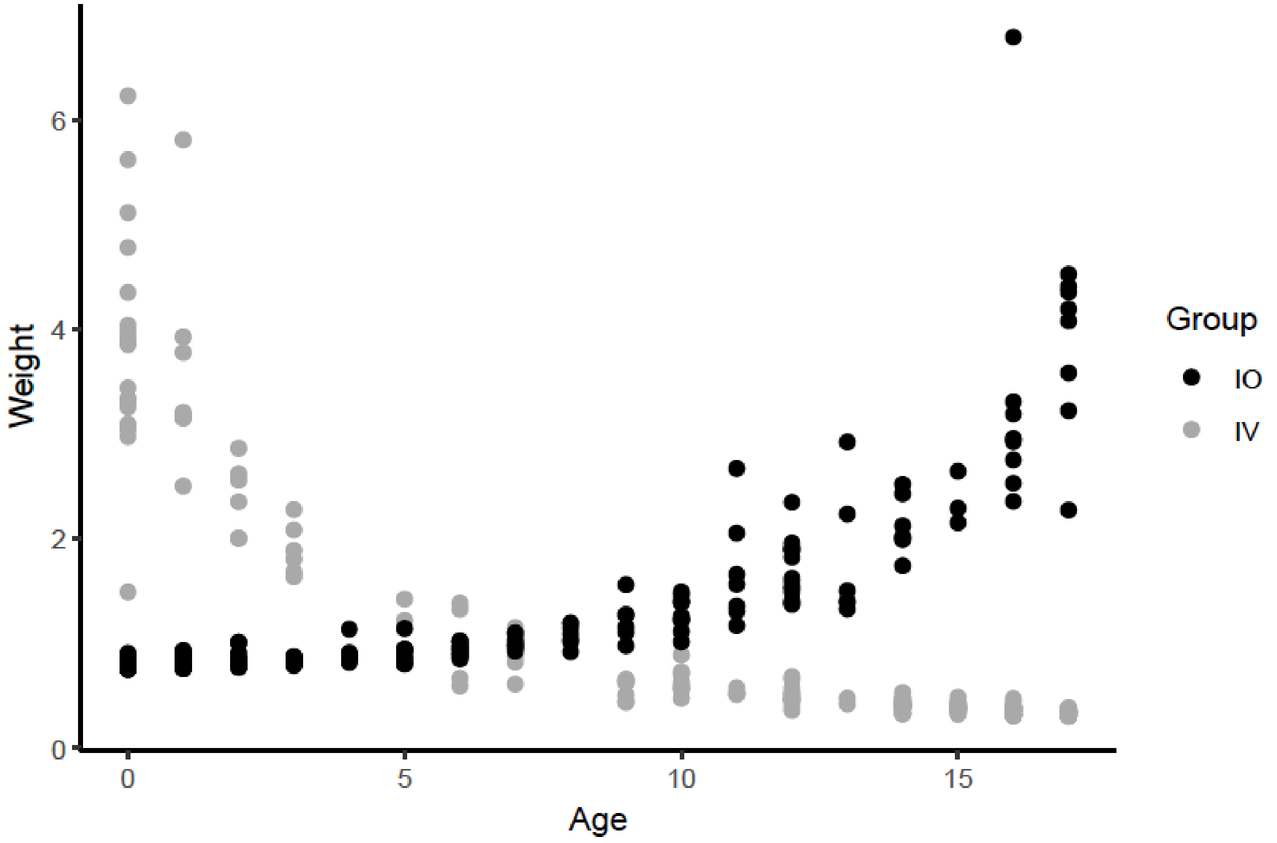

© 2025 Okubo M et al. *JAMA Network Open*.

eFigure 4. Cumulative proportion of patients without return of spontaneous circulation over time from epinephrine administration (A) and advanced life clinician arrival (B) in the weighted population, among who were younger than 1 year, stratified by the route of epinephrine administration.
IO, Intraosseous; IV, Intravenous.
Supplemental Figure 4A. Log-rank p value=0.86. The hazard ratio (95% confidence interval) for the IO group was 1.18 (0.18-7.66).
Supplemental Figure 4B. Log-rank p value=0.74. The hazard ratio (95% confidence interval) for the IO group was 1.38 (0.21-9.12).

Supplemental Figure 4A

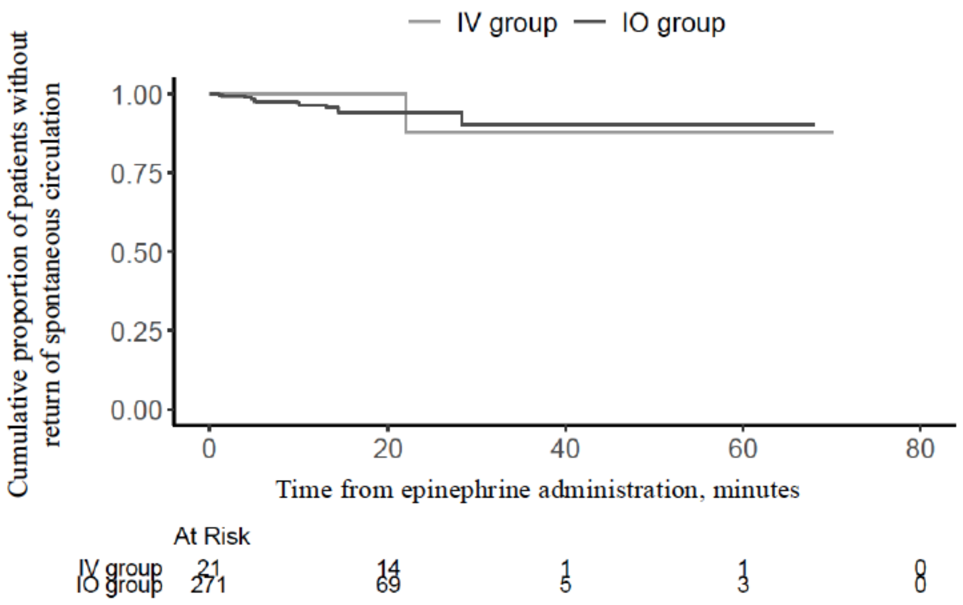

© 2025 Okubo M et al. *JAMA Network Open*.

Supplemental Figure 4B

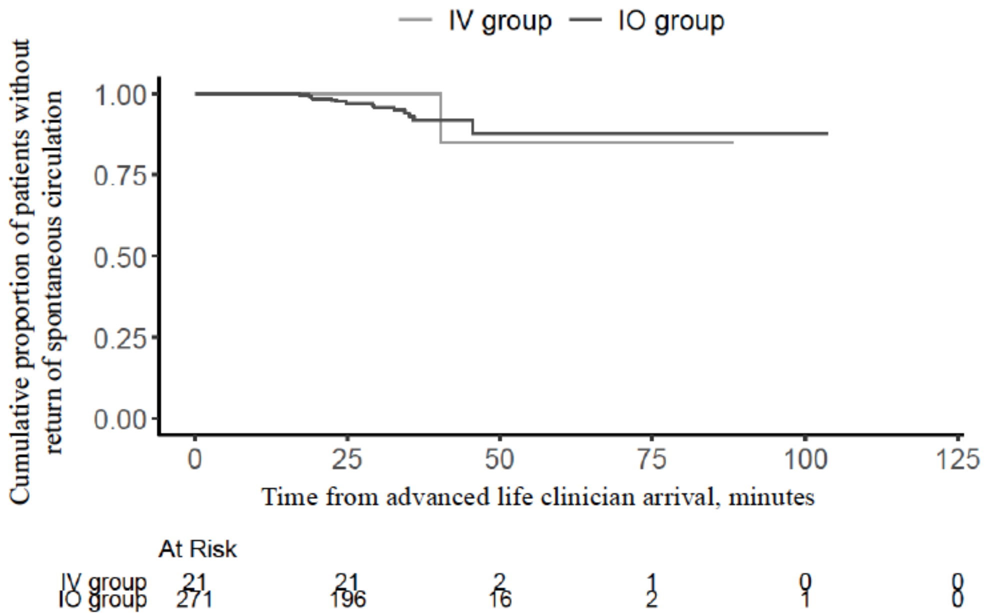

© 2025 Okubo M et al. *JAMA Network Open*.

eFigure 5. Cumulative proportion of patients without return of spontaneous circulation over time from epinephrine administration (A) and advanced life clinician arrival (B) in the weighted population, among who were older than or equal to 1 year and younger than or equal to 9 years, stratified by the route of epinephrine administration.
IO, Intraosseous; IV, Intravenous.
Supplemental Figure 5A. Log-rank p value=0.047. The hazard ratio (95% confidence interval) for the IO group was 0.39 (0.16-0.95).
Supplemental Figure 5B. Log-rank p value=0.16 The hazard ratio (95% confidence interval) for the IO group was 0.48 (0.21-1.10).

Supplemental Figure 5A

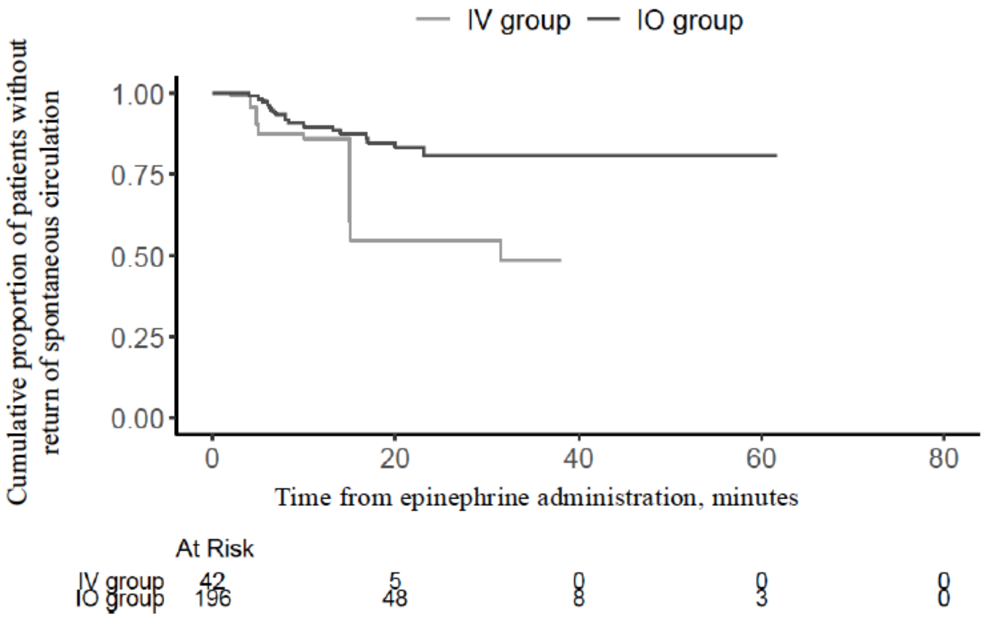

© 2025 Okubo M et al. *JAMA Network Open*.

Supplemental Figure 5B

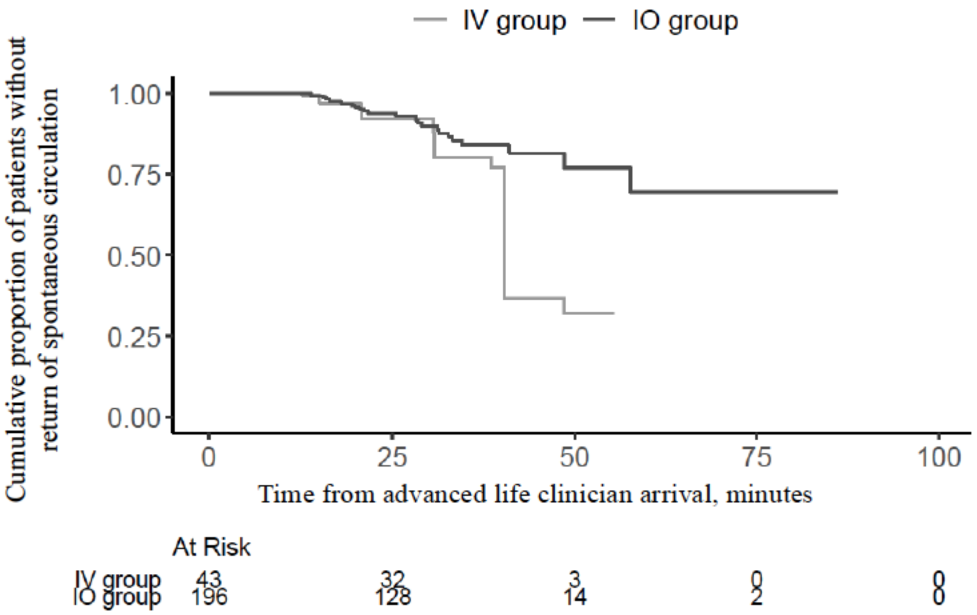

© 2025 Okubo M et al. *JAMA Network Open*.

eFigure 6. Cumulative proportion of patients without return of spontaneous circulation over time from epinephrine administration (A) and advanced life clinician arrival (B) in the weighted population, among who were older than or equal to 10 years, stratified by the route of epinephrine administration.
IO, Intraosseous; IV, Intravenous.
Supplemental Figure 5A. Log-rank p value=0.07. The hazard ratio (95% confidence interval) for the IO group was 0.58 (0.33-1.04).
Supplemental Figure 5B. Log-rank p value=0.15 The hazard ratio (95% confidence interval) for the IO group was 0.65 (0.36-1.18).

Supplemental Figure 6A

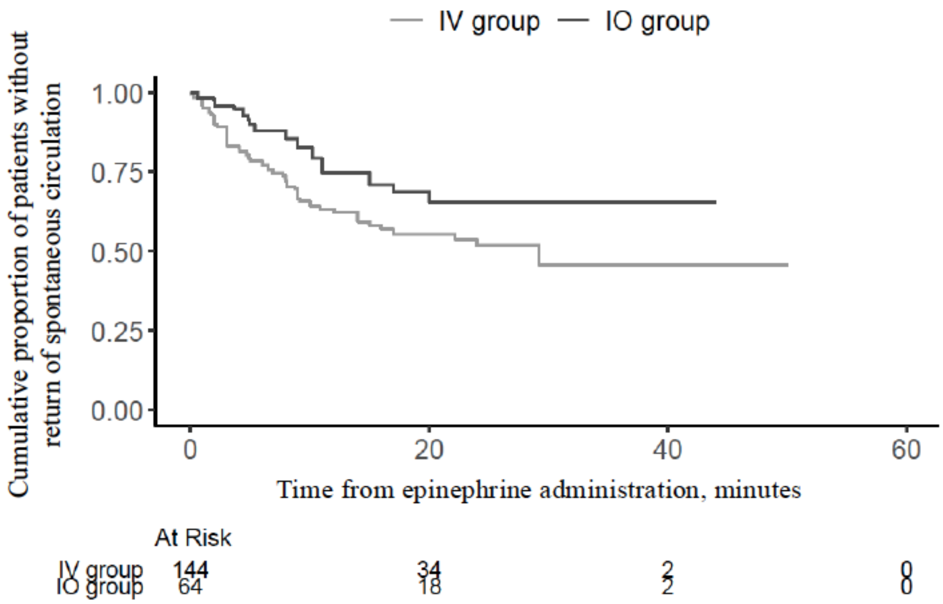

© 2025 Okubo M et al. *JAMA Network Open*.

Supplemental Figure 6B

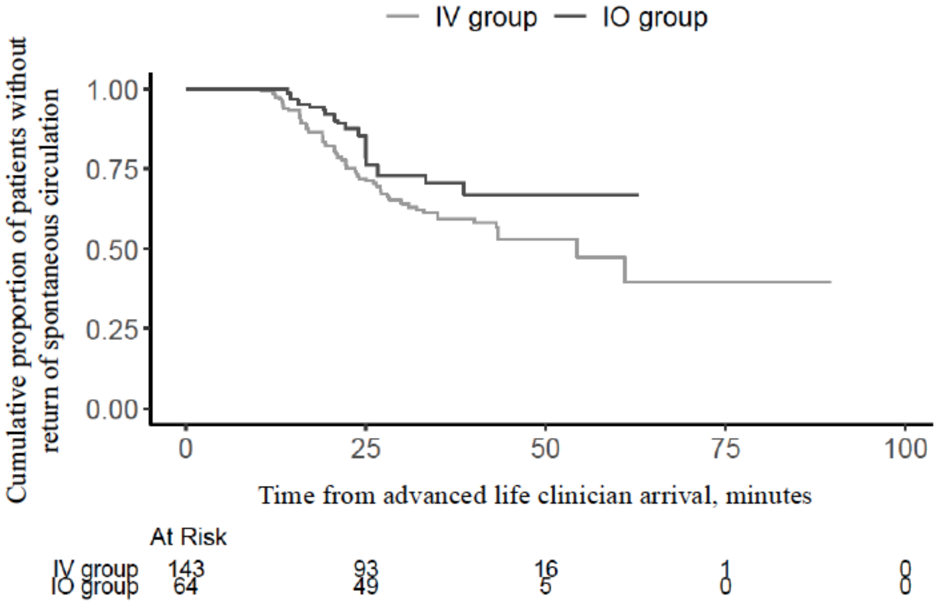

© 2025 Okubo M et al. *JAMA Network Open*.

Supplemental Figure 7. Cumulative proportion of patients without return of spontaneous circulation over time from epinephrine administration (A) and advanced life clinician arrival (B) in the weighted population including those who received epinephrine with either failed IO or IV access, stratified by the route of epinephrine administration.
IO, Intraosseous; IV, Intravenous.
Supplemental Figure 7A. Log-rank p value=0.25. The hazard ratio (95% confidence interval) for the IO group was 0.70 (0.43-1.15).
Supplemental Figure 7B. Log-rank p value=0.33 The hazard ratio (95% confidence interval) for the IO group was 0.75 (0.46-1.20).

Supplemental Figure 7A

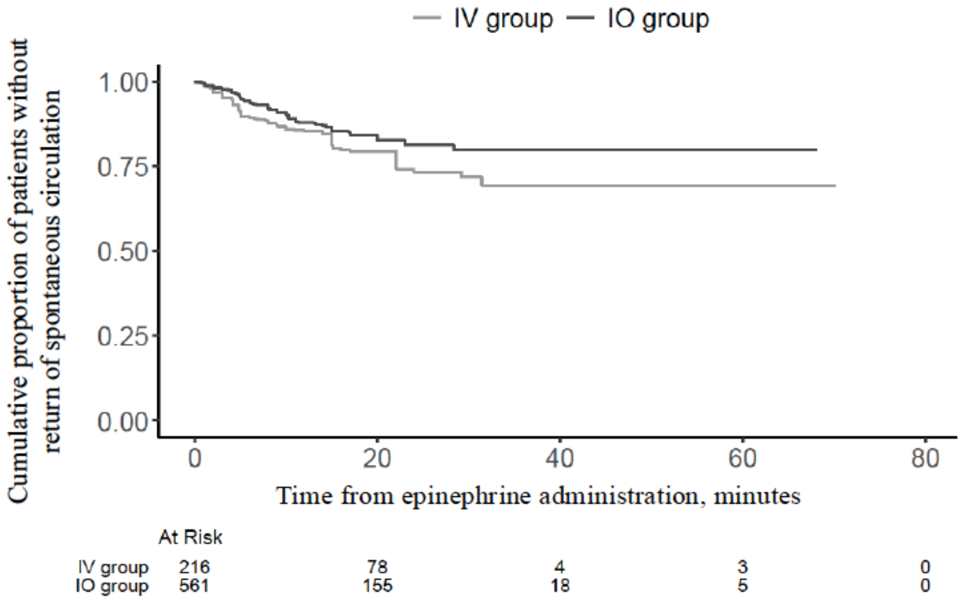

© 2025 Okubo M et al. *JAMA Network Open*.

Supplemental Figure 7B

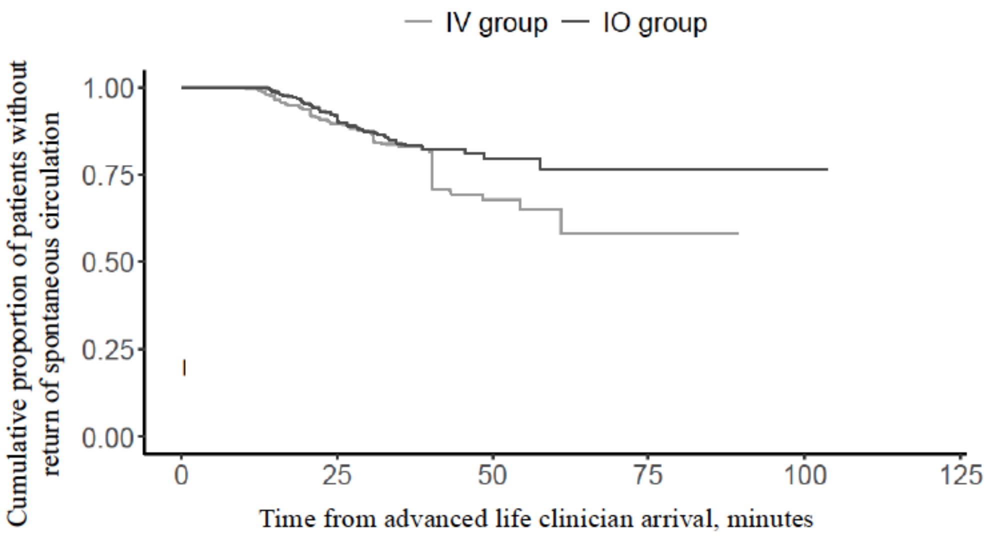

At Risk

IV group	214	173	24	3	0	0
IO group	560	400	41	5	1	0

© 2025 Okubo M et al. *JAMA Network Open*.
