# Supplementary material for: Intraosseous vs Intravenous Access for Epinephrine in Pediatric Out-of-Hospital Cardiac Arrest
Source: JAMA Netw Open. 2025 Jun 25;8(6):e2517291. doi: 10.1001/jamanetworkopen.2025.17291 (PMC12199053; doi:10.1001/jamanetworkopen.2025.17291)
Supplement: Supplement 2. — Data Sharing Statement [file jamanetwopen-e2517291-s002.pdf]

Data Sharing Statement

Okubo. Intraosseous vs Intravenous Access for Epinephrine in Pediatric Out-of-Hospital Cardiac Arrest. *JAMA Netw Open*. Published June 25, 2025.
doi:10.1001/jamanetworkopen.2025.17291

Data
Data available: Yes
Data types: Deidentified participant data, Data dictionary
How to access data: The de-identified Resuscitation Outcomes Consortium Epidemiologic Registry dataset is publicly available at the National Heart, Lung, and Blood Institute Biologic Specimen and Data Repository Information Coordinating Center (https://biolincc.nhlbi.nih.gov/home/).
When available: With publication

Supporting Documents
Document types: None

Additional Information
Who can access the data: Researchers whose proposed use of the data has been approved by the National Heart, Lung, and Blood Institute Biologic Specimen and Data Repository Information Coordinating Center.
Types of analyses: Any purpose.
Mechanisms of data availability: After approval of a proposal by the National Heart, Lung, and Blood Institute Biologic Specimen and Data Repository Information Coordinating Center.
